# TALEN‐mediated targeted mutagenesis of more than 100 *COMT* copies/alleles in highly polyploid sugarcane improves saccharification efficiency without compromising biomass yield

**DOI:** 10.1111/pbi.12833

**Published:** 2017-11-18

**Authors:** Baskaran Kannan, Je Hyeong Jung, Geoffrey W. Moxley, Sun‐Mi Lee, Fredy Altpeter

**Affiliations:** ^1^ Agronomy Department IFAS, University of Florida Gainesville FL USA; ^2^ Novozymes North America Inc Franklinton NC USA; ^3^ Clean Energy Research Center Korea Institute of Science and Technology (KIST) Seoul South Korea; ^4^ Plant Molecular and Cellular Biology Program IFAS, University of Florida Gainesville FL USA; ^5^ Genetics Institute University of Florida Gainesville FL USA; ^6^ Present address: Center for Natural Products Convergence Research Korea Institute of Science and Technology (KIST) Gangneung Gangwon‐do South Korea

**Keywords:** TALEN, field performance, genome editing, sugarcane, COMT, lignin, biofuel

## Abstract

Sugarcane is the world's most efficient feedstock for commercial production of bioethanol due to its superior biomass production and accumulation of sucrose in stems. Integrating first‐ and second‐generation ethanol conversion processes will enhance the biofuel yield per unit area by utilizing both sucrose and cell wall‐bound sugars for fermentation. RNAi suppression of the lignin biosynthetic gene *caffeic acid O‐methyltransferase* (*COMT*) has been demonstrated to improve bioethanol production from lignocellulosic biomass. Genome editing has been used in a number of crops for creation of loss of function phenotypes but is very challenging in sugarcane due to its highly polyploid genome. In this study, a conserved region of *COMT* was targeted with a single‐transcription activator‐like effector nuclease (TALEN) pair for multi‐allelic mutagenesis to modify lignin biosynthesis in sugarcane. Field‐grown TALEN‐mediated *COMT* mutants showed up to 19.7% lignin reduction and significantly decreased syringyl to guaiacyl (S/G) ratio resulting in an up to 43.8% improved saccharification efficiency. Biomass production of *COMT* mutant lines with superior saccharification efficiency did not differ significantly from the original cultivar under replicated field conditions. Sanger sequencing of cloned *COMT* amplicons (1351–1657 bp) revealed co‐editing of 107 of the 109 unique *COMT* copies/alleles in vegetative progeny of line CB6 using a single TALEN pair. Line CB6 combined altered cell wall composition and drastically improved saccharification efficiency with good agronomic performance. These findings confirm the feasibility of co‐mutagenesis of a very large number of target alleles/copies for improvement in crops with complex genomes.

## Introduction

Sugarcane is considered a prime feedstock for sustainable biofuel production due to its high biomass yield and ratooning ability (Byrt *et al*., 2011). The first‐generation biofuel conversion process utilizes sucrose, extracted from sugarcane stems with roller mills, for bioethanol production by a direct fermentation process. This process uses the abundant lignocellulosic sugarcane biomass residue, also called bagasse, for generation of electricity (Tew and Cobill, 2008). More value can be captured from this abundant resource by second‐generation conversion processes, which use pretreatment of biomass and enzymatic hydrolysis for the saccharification of the cell wall‐bound cellulose and hemicellulose (Naik *et al*., 2010; Vermerris *et al*., 2007). Thus, the integration of first‐ and second‐generation conversion technology should have environmental and economic benefits (Joelsson *et al*., 2016; Somerville *et al*., 2010) and is currently explored for commercial production of sugarcane‐derived bioethanol in Brazil (Dias *et al*., 2014).

Lignocellulosic sugarcane biomass consists of cellulose, hemicellulose, and lignin (Karp and Shield, 2008). Lignin is a recalcitrance factor for biofuel production from lignocellulosic biomass. Lignin prevents the accessibility of cellulose microfibrils by cellulase enzyme as well as adsorbs hydrolytic enzymes; thereby, it inhibits the release of cell wall‐bound sugars (Chen and Dixon, 2007; Weng *et al*., 2008). The lignin polymer consists of *p*‐hydroxyphenyl (H), guaiacyl (G), and syringyl (S) monomers (Li *et al*., 2008).

Conversion of 5‐hydroxyconiferyl alcohol to sinapyl alcohol, which is a component of the S subunit, is mediated by *caffeic acid O‐methyl transferase* (*COMT*) via the phenylpropanoid pathway (Hisano *et al*., 2009; Humphreys and Chapple, 2002). RNAi suppression or a knockout mutation of *COMT* resulted in reduction in both total lignin content and S/G ratio. Altered lignin content and composition following *COMT* suppression/mutagenesis were demonstrated in biofuel and/or forage crops like sugarcane (Jung and Altpeter, 2016; Jung *et al*., 2012, 2013), switchgrass (Baxter *et al*., 2014; Fu *et al*., 2011; Samuel *et al*., 2014), corn (Piquemal *et al*., 2002), sorghum (Saballos *et al*., 2008; Sattler *et al*., 2012), tall fescue (Chen *et al*., 2004), and alfalfa (Chen and Dixon, 2007; Guo *et al*., 2001).

Although lignin reduction by targeted genome editing may reduce costs associated with regulatory approval (Wolt *et al*., 2016), the polyploid nature of sugarcane genome creates challenges for this approach. Modern sugarcane cultivars have a genome size of approximately 10 Gb, large number (100–130) of chromosomes and ~12 homo(eo)logs at each locus resulting in a high level of genetic redundancy (Le Cunff *et al*., 2008; Piperidis *et al*., 2010; de Setta *et al*., 2014). The recent assembly of the draft sugarcane genome (Riaño‐Pachón and Mattiello, 2017) will accelerate targeted crop improvement. However, the complex architecture of its polyploid genome will remain a challenge for these efforts (Okura *et al*., 2016; Souza *et al*., 2011).

Transcription activator‐like effector nuclease (TALEN) is a genome‐editing tool enabling precise genome modifications, such as targeted mutagenesis, gene replacement, or insertion (Gurushidze *et al*., 2014; Li *et al*., 2012; Zhang *et al*., 2013). Targeted mutagenesis with TALEN has been successful in number of crops for creation of loss of function genotypes (reviewed in Baltes and Voytas, 2015; Weeks *et al*., 2016). Recently, TALEN‐mediated targeted mutagenesis in a highly polyploid sugarcane was reported (Jung and Altpeter, 2016), including generation of events with integration and expression of TALEN, evidence of targeted mutagenesis by capillary electrophoresis and sequence analysis of 89–148 nt PCR amplicons encompassing the TALEN target site and phenotypes, which displayed reduced lignin and altered lignin and cell wall composition under glasshouse conditions. Here, we report for the first time evaluation of TALEN‐mediated *COMT* mutant sugarcane lines in replicated field plots for agronomic performance, lignin content, lignin, and cell wall composition as well as saccharification efficiency. We also carried out Sanger sequencing of cloned 1351‐ to 1657‐bp‐long PCR amplicons. This allows now to report the number of co‐mutated *COMT* targets, resulting in improved saccharification efficiency in field‐grown sugarcane plants without compromising agronomic performance.

## Results

Generation of sugarcane with integration of COMT‐TALEN was reported by Jung and Altpeter (2016) and is briefly summarized in the Data S1.

### Number of *COMT* copies/alleles, mutation frequency, and types

Sequencing of 1351‐ to 1657‐bp‐long PCR amplicons of genomic DNA from *COMT* mutant line CB6 was used to determine number of *COMT* copies/alleles in the sugarcane genome. A total of 440 *COMT* amplicon containing plasmid colonies were sequenced by the Sanger method, and chromatograms were visually inspected to select 389 high‐quality Sanger read pairs with overlapping ends. Of these 389 high‐quality reads, 109 unique reads representing different *COMT* copies/alleles were identified (Table 1). Of the 109 unique reads, two reads displayed WT sequence in the targeted mutation site, while 107 reads displayed nucleotide insertions or deletions (InDels) in the TALEN target site, which indicates a mutation frequency of 98%. Five different exon variants, outside of the targeted mutation site, and 104 different intron variants were identified (Table S1). Identical target site mutations were confirmed in many different *COMT* copies/alleles. Different *COMT* copies/alleles were associated with intron sequence variations like InDels resulting in variations of intron length from 865 nucleotides to 1171 nucleotides, as well as sequence variations (single‐nucleotide polymorphisms, SNPs) in introns of the same size. Sequence reads representing different target site mutations within the same *COMT* copy/allele were also identified. A total of six unique reads, representing 5.6% of all unique reads, displayed more than one mutation type (up to 3; Table S1). Four of them had 7‐ and 48‐bp deletions, one of them had 4‐, 36‐, and 48‐bp deletions, and one of them displayed 3‐ and 10‐bp deletions.

**Table 1:** Number of *COMT* variants in sugarcane line CB6

Genotype	Number of quality reads	Number of quality reads with targeted mutation	Number of unique reads with WT sequence	Total number of unique reads with variation in exon and intron outside of targeted mutation site
*COMT* mutant	389	387	2	109

### Growth performance of TALEN‐mediated *COMT* mutant lines in the replicated field plots

**Figure 1:**
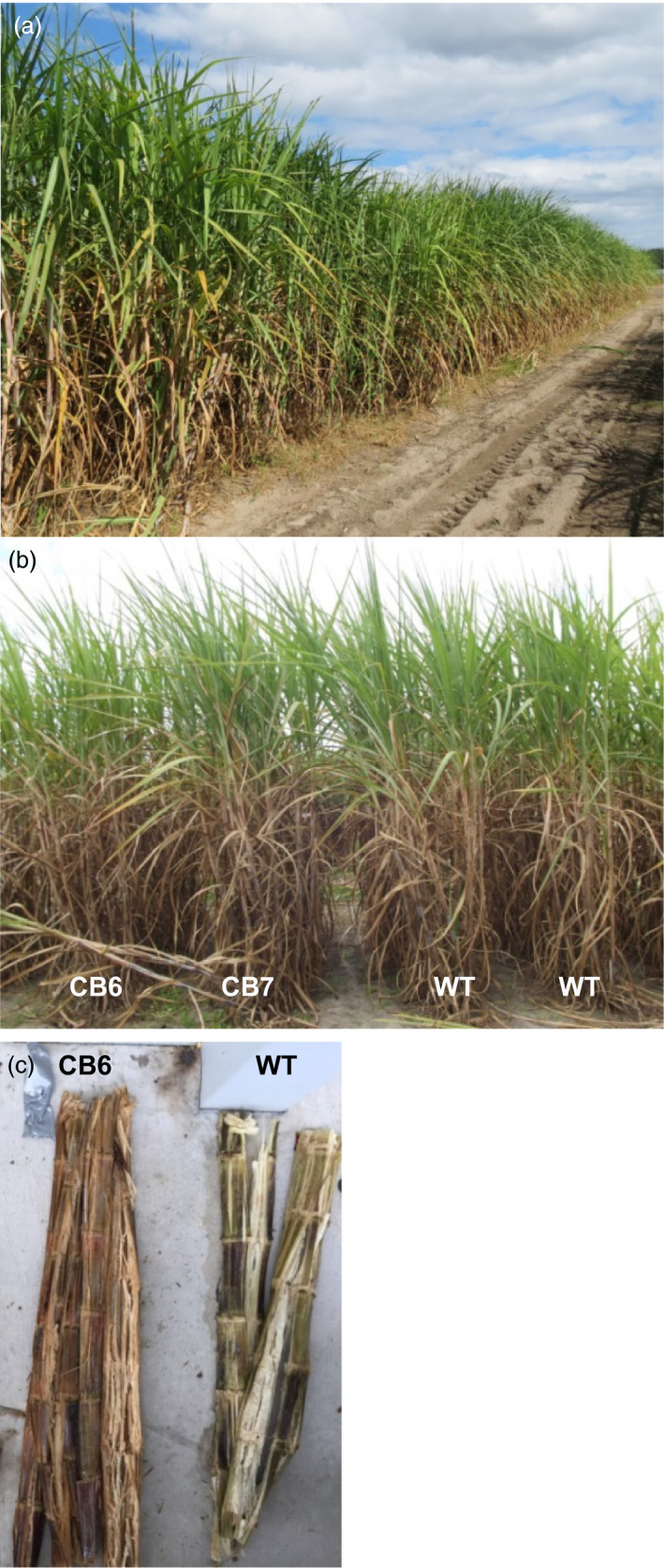
(a) Field trial of TALEN‐mediated *COMT* mutants. (b) Field performance of *COMT* mutant lines and wild type (WT). CB6 and CB7 are mutant lines derived through direct embryogenesis and biolistic transformation. (c) Stems of field‐grown TALEN‐mediated *COMT* mutant (CB6) in comparison with wild type (WT), immediately after juice extraction with roller mills.

The events evaluated in replicated field plots for biomass yield and agronomic traits included eight TALEN‐mediated *COMT* mutant lines including CB3‐8 derived from biolistic gene transfer and direct embryogenesis and CA4 and CA17 derived from *Agrobacterium* mediated gene transfer and indirect embryogenesis. In addition, control plants including the original cultivar (wild type; WT), callus‐derived control harboring the *npt*II gene (TC1), a callus‐derived non‐transgenic control (TC2), a direct embryogenesis‐derived transgenic control with no mutation (TC3), and a line with *COMT* suppression by RNAi (B401) were evaluated in these field plots (Table 2 and Figure 1). The plant height of *COMT* mutant lines varied from 174.4 cm (CB3) to 195.3 cm (CB5). However, these variations were not significantly different from WT (181.8 cm). Number of tillers produced by *COMT* mutant lines was non‐significantly higher than WT (8.2) except CA4 and CA17, which displayed significantly more tillers. Stalk diameter varied from 19.3 mm (TC1 and TC2) to 22.7 mm (CB5). Among the *COMT* mutant lines, CA17 had significantly thinner stalks (15.9 mm) than WT (21.4 mm). Dry biomass yield of *COMT* mutant lines, transgenic, and tissue culture controls was not significantly different from WT (36.0 t/ha) with the exception of line CA17 and control TC2, which displayed a reduced biomass yield. Both, CA17 and TC2, were regenerated from callus while all CB lines as well as control TC3 were derived from direct embryogenesis. There was a trend to elevated biomass production in several of the *COMT* mutants with CB5 producing 43.7 t/ha, followed by CB7 with 40.4 t/ha and CB8 with 36.5 t/ha. Lines with a trend to elevated biomass displayed a reduction in soluble solids/Brix units by 17%–19% compared to WT with no significant difference in juice volume. Line CB6 resembled WT with no significant difference in any of the phenotypic data.

**Table 2:** Phenotypic performance of *COMT* mutants and controls under field conditions

Line	Plant height (cm)	No. of tillers per plant	Stalk diameter (mm)	Biomass yield (t/ha)	Juice volume (mL/100 g of fresh stalks)	Soluble solids (° Brix)
WT	181.8	8.2	21.4	36.0	37.1	21.0
TC1	153.0*	11.9*	19.3	29.7	40.6	20.2
TC2	177.0	9.2	19.3	23.6*	40.1	20.5
TC3	181.2	8.2	22.2	32.0	39.3	21.1
CB3	174.4	9.2	19.5	32.0	32.0	18.7
CB4	182.3	8.8	21.3	35.3	34.0	20.0
CB5	195.3	9.2	22.7	43.7	32.6	17.5*
CB6	186.8	9.0	20.0	35.4	29.7	20.4
CB7	190.8	8.8	21.4	40.4	31.1	17.4*
CB8	189.7	9.1	21.5	36.5	32.7	17.0*
CA4	186.3	10.4*	19.5	31.7	30.4	16.6*
CA17	191.9	10.8*	15.9*	24.0*	30.5	16.4*
B401	173.8	11.8*	21.7	36.8	42.9	21.3

WT, wild‐type sugarcane; TC1, callus‐derived control harboring the *npt*II gene; TC2, callus‐derived non‐transgenic control; TC3, direct embryogenesis‐derived transgenic control with no mutation; CB3‐CB8, transgenic lines derived from biolistic transformation; CA4‐CA17, transgenic lines derived from *Agrobacterium*‐mediated transformation; B401, *COMT* RNAi line.

Values with asterisk in the same column indicate significant difference compared to WT (*n* = 3, *P *<* *0.05) as determined by *t*‐test.

### *COMT* expression in COMT RNAi and TALEN‐mediated *COMT* mutant lines

Quantitative real‐time RT‐PCR analysis was performed to quantify *COMT* gene expression in *COMT* mutant or RNAi‐suppressed plants compared to WT plants (Figure S1). *COMT* expression in RNAi line was 41.8% for B401, which corresponds to 58% *COMT* gene suppression (Figure S1). The *COMT* expression in TALEN‐mediated *COMT* mutant lines varied from 26.7% (CA4) to 91.4% (CB3) of WT transcripts.

### Mutation frequency and lignin modification due to TALEN‐mediated *COMT* mutation

**Figure 2:**
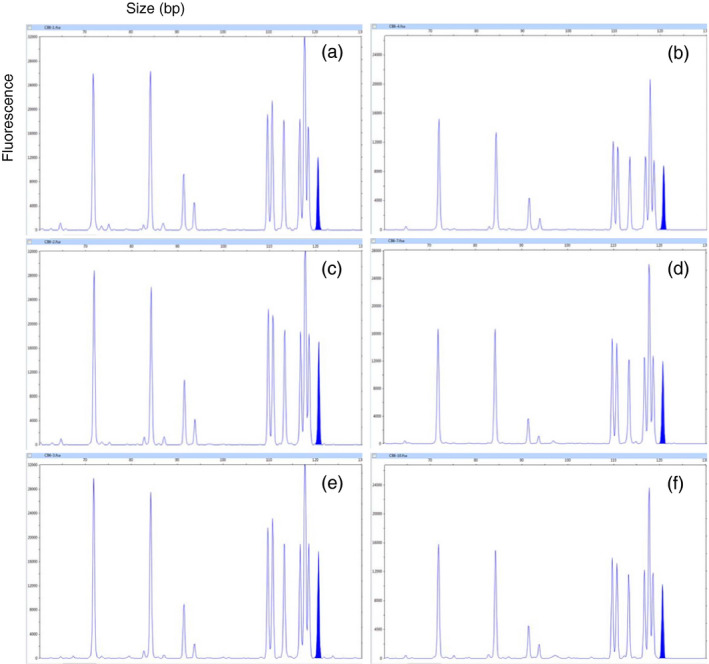
Representative electropherogram for different tillers and different plants of *COMT* mutant line CB6. (a,b) Two different tillers of the same plant in replication 1. (c,d) Two different tillers of the same plant in replication 2. (e,f) Two different tillers of the same plant in replication 3. Peak representing unmodified *COMT* is highlighted in blue.

Mutation frequencies of field‐grown *COMT* mutant lines were determined by capillary electrophoresis (CE) analysis (Table 3). Mutation frequency ranged from 51.4% (CB4) to 92.5% (CB6). Replicated field samples of mutant lines displayed uniform CE mutation pattern despite variations in the relative fluorescence (Figure 2). WT plants displayed no mutation and its estimated lignin content was 241.8 mg/g of DW. The amount of lignin reduction with respect to WT varied from 10.9% reduction in CB5 to 19.7% in CB7. Lignin reduction did not correlate with mutation frequencies of field‐grown plants. *COMT* mutant lines with high biomass production were used for the estimation of lignin monomers such as syringyl (S) and guaiacyl (G). The S/G ratio of WT was 0.91, whereas CB5, CB6, and CB7 displayed reduced S/G ratios of 0.80, 0.63, and 0.71, respectively (Table 3).

**Table 3:** Mutation frequency determination of TALEN‐mediated lignin reduction lines under field conditions

Line	Mutation frequency (%)^a^	AcBr Lignin content (mg/g DW)^b^	Lignin reduction (%)	S/G molar ratio^c^
WT	0.0	241.8 ± 1.3	–	0.91
TC3	0.0	241.2 ± 4.2	0.2	0.89
CB3	70.0	200.6 ± 2.6*	17.0	n.a.
CB4	51.4	209.4 ± 5.3*	13.4	n.a.
CB5	69.9	215.5 ± 4.5*	10.9	0.80*
CB6	92.5	194.6 ± 6.1*	19.5	0.63*
CB7	68.1	194.1 ± 2.0*	19.7	0.71*
CB8	71.7	197.7 ± 5.9*	18.2	n.a.
CA4	80.0	198.9 ± 5.8*	17.7	n.a.
CA17	89.3	195.9 ± 4.3*	19.0	0.47*
B401	n.a.	219.0 ± 6.3*	9.4	n.a.

WT, wild‐type sugarcane; TC3, direct embryogenesis‐derived transgenic control with no mutation; CB3‐CB8, transgenic lines derived from biolistic transformation; CA4‐CA17, transgenic lines derived from *Agrobacterium*‐mediated transformation; B401, *COMT* RNAi line; DW, dry weight; n.a., not analyzed.

Values with asterisk in the same column indicate significant difference compared to WT (*n* = 3, *P *<* *0.05) as determined by *t*‐test.

a Mutation frequency estimated by relative fluorescent quantitation based on capillary electrophoresis electropherogram. Mutation frequency (%) = (Sum of peak height of all mutant peaks over sum of peak height of all peaks including wild‐type peak) × 100.

b Acetyl Bromide (AcBr) method was used to determine total lignin content.

c Ratio of monolignol compositions of S (syringyl) and G (guaiacyl) subunits and expressed as mg/g DW.

### Effect of TALEN‐mediated mutation on cell wall carbohydrates

The composition of cell wall carbohydrates was evaluated in *COMT* mutants, RNAi‐suppressed COMT line, and WT sugarcane plants (Table 4). The amount of glucose in the plant cell wall of *COMT* mutant or RNAi‐suppressed COMT sugarcane did not differ from that of WT. TALEN‐mediated *COMT* mutant lines CA17 and CB6 displayed a significant increase in both xylose and arabinose contents as compared to WT by 3.4%–7.6% and 19.2%–22.5%, respectively (Table 4).

**Table 4:** Cell wall carbohydrates in *COMT* suppressed or mutant sugarcane lines

Line	Cell wall carbohydrates (mg/g DW)			Total sugar
	Glucose	Xylose	Arabinose	
WT	437.3 ± 3.0	193.2 ± 1.1	24.5 ± 0.5	655.0
TC3	433.0 ± 6.9	189.1 ± 11.3	26.0 ± 0.7	648.1
B401	427.9 ± 7.7	194.0 ± 0.3	29.5 ± 0.1*	651.4
CA17	434.2 ± 5.9	199.8 ± 2.4*	30.0 ± 0.9*	664.0
CB5	437.6 ± 6.8	195.8 ± 3.6	22.7 ± 1.7 *	656.1
CB6	434.7 ± 6.1	208.5 ± 3.4*	29.2 ± 0.5*	672.4
CB7	435.2 ± 0.2	185.4 ± 1.7*	27.3 ± 0.6*	647.9

WT, wild‐type sugarcane; TC3, direct embryogenesis‐derived transgenic control with no mutation; CB5, CB6, and CB7, transgenic lines derived from biolistic transformation; CA17, transgenic lines derived from *Agrobacterium*‐mediated transformation; B401, *COMT* RNAi line; DW, dry weight.

Values with asterisk in the same column indicate significant difference compared to WT (*n* = 3, *P *<* *0.05) as determined by *t*‐test.

### Improvement in saccharification efficiency by *COMT* mutation

**Figure 3:**
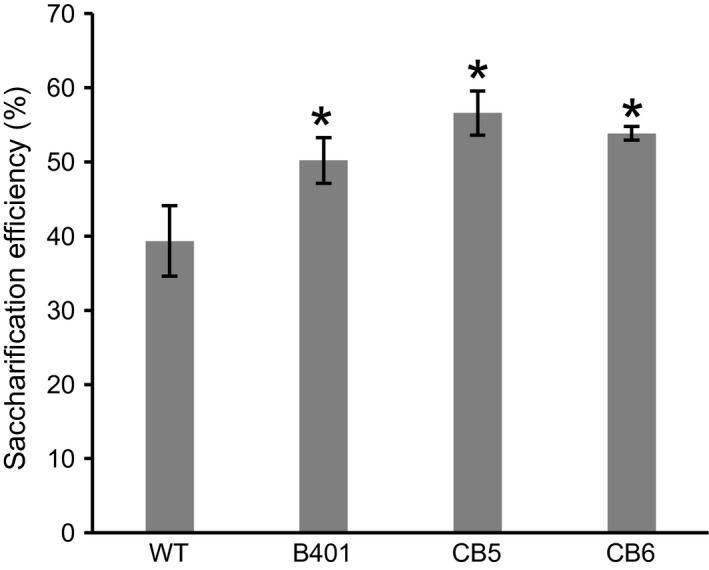
Saccharification efficiency of lignocellulosic biomass into directly fermentable glucose. Wild‐type sugarcane (WT); sugarcane with RNAi suppression of *COMT* (B401), TALEN‐mediated *COMT* mutant sugarcane lines (CB5, CB6). Error bars indicate standard error (*n* = 2). Asterisk above the bars indicates significant difference from WT at *P *<* *0.05 in *t*‐test.

Glucose yield from lignocellulosic biomass of *COMT* mutant or RNAi‐suppressed COMT lines was determined by enzymatic hydrolysis with dilute acid pretreatment. *COMT* mutant sugarcane displayed significantly improved glucose yield from lignocellulosic biomass by up to 44% compared to WT. The saccharification efficiencies were 39%, 50%, 57%, and 54% for WT, B401, CB5, and CB6, respectively (Figure 3).

### Effect of lignin modification on resistance to disease and insects and lodging

No significant differences were observed between *COMT* mutated/suppressed lines and WT for orange rust or red lesion on midrib (Table S2). qRT‐PCR analysis indicated the absence of sugarcane yellow virus (SCYLV) infection in *COMT* mutant or WT plants (Table S2). Lodging was absent from all entries at the time of harvest (Figure 1a), and therefore, data are not shown.

## Discussion

Designer nucleases like TALEN, zinc finger nucleases, meganucleases, CRISPR/Cas, or Cpf are key to enabling site‐directed genome modifications by targeting double‐stranded DNA breaks in genes of interest. Refinement of these genome‐editing tools have enabled unprecedented precision in genetic modifications, revolutionizing functional genomics and crop improvement (Altpeter *et al*., 2016; Hilscher *et al*., 2017; Voytas and Gao, 2014). Data from field experiments revealing the agronomic performance of genome‐edited crops are just emerging (Shi *et al*., 2017). Here, we present the first report of agronomic and/or conversion performance of a crop, which underwent genome editing with TALEN. Sugarcane is highly polyploid (*x* = 10–13), interspecific hybrid with chromosome number 2*n* = 100–130 and the genome size of ~10 Gb (Parthasarathy, 1948; Brandes and Artschwager, 1958; Piperidis *et al*., 2010; de Setta *et al*., 2014). This highly redundant genomic context requires extensive co‐editing of a large number of targets for creation of a ‘loss of function’ phenotype. Our most remarkable finding is that the extensive co‐editing of more than 100 copies/alleles of the lignin biosynthetic gene *caffeic acid O‐methyltransferase* (*COMT*) did not compromise agronomic performance under field conditions. However, the targeted mutagenesis of the vast majority of the more than 100 *COMT* copies/alleles improved saccharification of cell wall‐bound sugars by 39%–44% for biofuel production. This was accompanied by significantly reduced lignin content and altered lignin monomer ratio.

*COMT* catalyzes O‐methylation of 5‐hydroxyconiferaldehyde and 5‐hydroxyconiferyl alcohol, diverting metabolic flux to formation of the syringyl (S) lignin monomer (Callazo *et al.,* 1992; Vignols *et al*., 1995; Bout and Vermerris, 2003). Altered lignin composition creates brown vascular tissue in the stem and leaves. Brown midrib mutants (*bmr*) were among the earliest described and characterized mutants in maize (Jorgenson, 1931; Kuc and Nelson, 1964; Vignols *et al*., 1995). In diploid crops like maize and sorghum, *bmr* mutants arise naturally or following chemical mutagenesis (Jorgenson, 1931; Porter *et al*., 1978). However, the genetic redundancy of the highly polyploid sugarcane genome prevents the generation of natural or chemically induced *bmr* mutants in this crop. RNAi targeted toward a highly conserved catalytic domain in the first exon of *COMT* reduced S/G monomer ratio and lignin content and resulted in improved saccharification efficiency in sugarcane (Jung *et al*., 2012, 2013). This result informed a strategy for TALEN‐mediated targeted mutagenesis of this highly conserved catalytic *COMT* domain (Jung and Altpeter, 2016). Based on short 454 amplicons of the *COMT* target region and capillary electrophoresis, an almost complete knockout of the target *COMT* alleles was reported in sugarcane along with reduction in lignin and S/G lignin monomer ratio (Jung and Altpeter, 2016). However, the total number of the mutated *COMT* copies could not be assessed with short amplicon sequencing, as the genome of the parental sugarcane cultivar CP88‐1762 has not been fully sequenced and due to the high conservation of the targeted first *COMT* exon. Here, we confirm that only four SNPs exist in the entire first exon. To reveal different *COMT* copies/alleles and account for the number of targeted *COMT* modifications, we included the first *COMT* intron as indicator of sequence variation in different alleles/copies in the sequence analysis. Therefore, 1351‐ to 1657‐bp‐long, cloned *COMT* amplicons spanning exon 1 encompassing the mutation target site, intron 1 and the 5′ region of exon 2, were sequenced using the Sanger method (Figures S2 and S3). The sequence alignment of 389 paired quality reads from cloned *COMT* amplicons of *bmr* line CB6 with good agronomic performance revealed 109 unique *COMT* copies/alleles with five exon variants outside the targeted mutation site and 104 intron variants (Tables 1 and S1). In sharp contrast, the haploid genome of sorghum (diploid species) a close relative of sugarcane contains only seven copies of the *COMT* gene. The drastically lower redundancy of *COMT* in sorghum allowed successful chemical mutagenesis for generation of *bmr* phenotypes in this crop (Bout and Vermerris, 2003; Porter *et al*., 1978).

A single TALEN pair targeted to the highly conserved catalytic domain in the first exon of COMT was able to co‐mutate 107 of the 109 identified *COMT* copies/alleles in sugarcane *bmr* line CB6 without compromising its agronomic performance. To our knowledge, the maximum number of genomic sites previously reported to be simultaneously edited has been 62 (Yang *et al*., 2015) using two CRISPR‐Cas9 gRNAs targeted to the highly conserved catalytic domain of porcine endogenous retrovirus polymerase genes (PERVs) in PK15 cells of pigs. However, the successful application of this cellular approach to animals is still pending. TALEN has been widely used for the improvement in agronomic traits in a variety of diploid and polyploid crops (Baltes and Voytas, 2015; Hilscher *et al*., 2017; Weeks *et al*., 2016). However, so far typically only a small number of co‐mutated target genes were reported in plants. For instance, in potato, Clasen *et al*. (2016) mutated four alleles/copies of the vacuolar invertase gene (*VInv*) using TALEN. Both *FAD2‐1A* and *FAD2‐1B* genes were co‐mutated by TALEN in soya bean (Haun *et al*., 2014). Wang *et al*. (2014) co‐mutated three copies of the *TaMLO* gene in hexaploid bread wheat with a single TALEN pair targeted to a conserved region in exon 2. Multiplexing of up to eight gRNAs in CRISPR/Cas9‐mediated targeted co‐mutagenesis of seven genes of the *OsFTL* gene family in rice (Ma *et al*., 2015).

The effective spacer length for cleavage by a TALEN pair is 12–21 nucleotides (Miller *et al*., 2011) or 13–16 bp for TALEN pairs with C‐terminal truncation (Christian *et al*., 2012). Both repeated cleavage by constitutively expressed TALENs and incomplete editing before the first division of the embryogenic cell that received the genome‐editing tool may result in a cellular mosaic pattern or chimerism (Jung and Altpeter, 2016; Zhang *et al*., 2014). Typical for such mosaic pattern is the observation that many of the same copies/alleles in different plant cells or vegetative progenies have different mutation frequencies or different mutation types. Six unique *COMT* reads from *bmr* mutant CB6 displayed more than one mutation type (up to 3), which represents only 5.6% of all unique reads that contain mutations (Table S1). These mutation type variants most likely arose due to progression of deletions until cleavage was terminated by insufficient spacer length. However, 98% of all sequence reads in bmr mutant CB6 were co‐mutated (Table 1). Capillary electrophoresis (CE) in multiple replications and tillers displayed identical peak patterns suggesting that the vast majority of the targeted *COMT* mutations have occurred before the first division of the embryogenic cell that received the genome‐editing tool. Mutation frequencies observed in the replicated field samples corresponded well to glasshouse‐grown *COMT* mutant lines reported earlier (Jung and Altpeter, 2016). Brown coloration was observed immediately after crushing or cutting of stems of all *COMT* mutants with significantly reduced lignin content and in contrast to tissue culture and transgenic controls and WT. Plants from the entire field experiment were crushed with a roller mill and all the crushed stems from *COMT* mutants with significantly reduced lignin content were instantly brown, suggesting the absence of chimerism in the mutated events and stability of the trait in the vegetative progenies (Figure 1c). Earlier, brown coloration in freshly cut stem tissues of transgenic sugarcane or switchgrass with RNAi suppression of *COMT* and lignin reduction was also reported (Fu *et al*., 2011; Jung *et al*., 2012).

Targeting the TALEN to the highly conserved catalytic domain of *COMT* may have contributed to a loss of function phenotype even in the case of silent mutations by deleting one or several essential amino acids for catalytic activity. This may explain the absence of a correlation between *COMT* expression level and observed cell wall modifications in *COMT* mutants. CB7 displayed a similar (19.7%) lignin reduction as CB6 (19.5%) as well as a similar reduction in S/G lignin monomer ratio. However, both lines displayed a different mutation frequency (68% vs 93%) according to CE analysis. This suggests that a large proportion of the *COMT* copies/alleles are either non‐functional, are expressed at a low level or differ in the mutation type. The abundant allelic *COMT* variation in sugarcane can now be further explored for targeted mutation of specific copies/alleles similar to the CRISPR/Cas9 approach reported for the *4CL* gene family in *Populus* (Zhou *et al*., 2015).

Identification of off‐target mutations is a daunting task in sugarcane, even with the recent assembly of the draft sugarcane genome (Riaño‐Pachón and Mattiello, 2017). TALEN appears to be superior to CRISPR/Cas9 in regard to on‐target mutagenesis (Shan *et al*., 2015). In the absence of genomewide analysis of off‐target mutagenesis, agronomic performance is the best indicator for unintended mutations that may arise by genome editing or somaclonal variation. All *COMT* mutant lines and transgenic controls, which underwent direct embryogenesis and biolistic gene transfer (CB3–CB8; TC3), resembled the original sugarcane cultivar (WT) in biomass yield and biomass related traits (Table 2). However, *COMT* mutant line CA17, which was derived from *Agrobacterium‐*mediated gene transfer and the tissue culture control TC2 which like CA17 regenerated plants following the longer tissue culture process of indirect embryogenesis, displayed significantly reduced biomass yields. This also suggests that minimizing somaclonal variation by reducing the time in tissue culture (Taparia *et al*., 2012) and/or by using biolistic instead of *Agrobacterium*‐mediated gene transfer (Joyce *et al*., 2014; Wu *et al*., 2015) may contribute to better agronomic performance. Callus‐derived sugarcane lines with RNAi suppression of *COMT* also displayed a moderate decrease in biomass yield compared to the original cultivar along with reduced stalk length and stalk diameter (Jung *et al*., 2013). Transgenic switchgrass with RNAi suppression of *COMT* and lignin reduction did not display any negative impact on biomass production or diseases susceptibility (Baxter *et al*., 2014).

*COMT* mutant sugarcane lines and controls did not display any lodging prior harvest (Figure 1a) and disease scores did not differ significantly from control plants in this study (Table S2). For a final conclusion on disease response, large‐scale and multisite field testing is required. Alternative targets beside *COMT* have been explored for suppression of the lignin biosynthetic pathway, and most of them compromise plant growth or impair the plant defense system (Bonawitz and Chapple, 2013; Chen and Dixon, 2007; Eudes *et al*., 2014). However, examples of lignin‐modified plants with increased resistance to pathogens due to accumulation of lignin precursors and other phenolic compounds were also reported (McKeehen *et al*., 1999; Quentin *et al*., 2009; Sattler and Funnell‐Harris, 2013).

Lignin is a major recalcitrance factor for saccharification of cell walls (Chen and Dixon, 2007; Li *et al*., 2008). Field‐grown *COMT* mutant lines with reduced lignin and S/G ratio displayed 39%–44% improvement in saccharification efficiency over the original sugarcane cultivar (Figure 3). This moderately exceeds the reported improvements in saccharification efficiency following RNAi suppression of *COMT* in switchgrass (17%–22%, Fu *et al*., 2011; 9%–34%, Baxter *et al*., 2014) and sugarcane (19%–32%, Jung *et al*., 2013). Reduction in total lignin content and S/G ratio observed in this experiment were consistent with earlier reports from RNAi suppression of *COMT* in switchgrass (Baxter *et al*., 2014; Fu *et al*., 2011; Samuel *et al*., 2014), sugarcane (Jung *et al*., 2012, 2013), maize (Piquemal *et al*., 2002), tall fescue (Chen *et al*., 2004), alfalfa (Chen and Dixon, 2007), poplar (Studer *et al*., 2011), and *bmr* mutant sorghum (Dien *et al*., 2009). RNAi in contrast to genome editing depends on continued expression of transgenes. In contrast, targeted mutagenesis by sequence‐specific nuclease is heritable over generations in the absence of transgene expression (Char *et al*., 2015; Gao *et al*., 2010; Wang *et al*., 2014).

Sugarcane is vegetatively propagated for commercial production to maintain the original genotype while avoiding the extensive segregation of the highly polyploid genome. Therefore, removing the genome‐editing tool by Mendelian segregation of mutant and transgene alleles as described for seeded crops (Xu *et al*., 2015) would compromise the agronomic performance of sugarcane. Alternatively, non‐transgenic approaches to genome editing that have recently been demonstrated in other model and crop systems and are currently explored for generation of genome‐edited sugarcane without transgene footprint. These include transient delivery of DNA, or delivery as non‐integrative molecules like RNA, protein, or ribonucleoprotein complex (Luo *et al*., 2015; Svitashev *et al*., 2016; Zhang *et al*., 2016).

### Conclusion

A single TALEN pair designed to target the highly conserved domain of *COMT* was able to mutate 98% of the more than 100 *COMT* copies/alleles as revealed by Sanger sequencing of cloned, long PCR amplicons. Field‐grown *COMT* mutants displayed improved saccharification efficiency of up to 44% along with reduced lignin content and S/G lignin monomer ratio with no significant difference in biomass production and agronomic performance compared to the original sugarcane cultivar (WT). These findings demonstrate the great potential of genome editing for crop improvement and will contribute to more efficient and sustainable biofuel production from sugarcane.

## Experimental procedures

Generation of TALEN‐mediated *COMT* mutant lines from sugarcane is described in Jung and Altpeter (2016) and is briefly summarized in the Data S1.

### Number of *COMT* copies/alleles in sugarcane genome

Line CB 6 was chosen for this analysis since it combined a high mutation frequency based on capillary electrophoresis and 454 sequencing (Jung and Altpeter, 2016) with excellent conversion and agronomic performance. Genomic DNA was isolated from leaf tissues of field‐grown *bmr* mutant line (CB6) using CTAB method (Murray and Thompson, 1980). Isolated DNA was quantified and 100 ng DNA was used as a template for PCR. A region of *COMT*, spanning exon 1 through exon 2 (~1600 bp), which encompasses mutation target site and intron (Figure S3), was amplified by Q5 High‐Fidelity DNA Polymerase (NEB, Ipswich, MA) under the following conditions: 98 °C for 30 s, 34 cycles of amplification at 98 °C for 10 s, 72 °C for 10 s, and 72 °C for 10 s, and final extension at 72 °C for 2 min using 4F and BK_RO7 primers (Table S3). PCR amplicons were separated in a 1.5% agarose gel electrophoresis and targeted gel fragment excised under UV light after ethidium bromide staining. Amplicons were purified from gel fragments using QIAquick kit (Qiagen) and ligated into pGEM^®^‐T easy vector using T4 DNA ligase (Promega, Madison, WI). The ligated plasmid containing insert was electroporated into competent *E. coli* cells (NEB) and grown at 37 °C for 16 h. Transformed colonies were harvested after blue‐white select screening, and DNA was prepared using GeneJET miniprep kit (Thermo Fisher Scientific Inc., Waltham, MA). Sanger sequencing was performed on both strands using M13F and M13R primers at the Eurofins Genomics (Huntsville, AL). Sequence chromatograms were visually checked for quality, and sequences were trimmed manually. Quality reads with overlapping ends following both M13F and M13R sequencing were aligned with the multiple sequence alignment tool CLUSTALW (Table S1).

### Phenotypic evaluation of *COMT* mutants in replicated field plots

For field testing, mature nodes of glasshouse‐grown *COMT* mutant lines (eight lines) and control plants (transgenic controls, tissue culture control, RNAi control, and WT) were planted in 3‐L pots containing Fafard No. 2 mix (Sun Gro Horticulture, FL) irrigated once a day and fertilized biweekly with Miracle‐Gro Lawn Food (Scotts Miracle‐Gro, Marysville, OH). Three vegetative tillers were randomly selected for each of the eight TALEN‐mutated lines. Each of the three vegetative progeny was used for planting a separate replication of the field plots. Five plants were planted for each of the three replications per line. Therefore, 15 uniformly grown plants were selected and transplanted for each of the eight TALEN lines and for each of the controls to the field plots on Mar 25, 2015 at the University of Florida, Plant Science Research and Education Unit (PSREU), Citra, Florida, USA, under USDA/APHIS permit 13‐299‐101r‐a1. The field plots were laid out in a randomized complete block design (RCBD) of single row plots per accession with three replications in loamy sand soil. Each single row plot represents five plants per replication of mutant line or control plants, and each replication was surrounded by one row of the original cultivar (WT) as border plants. The spacing between rows and plants within each row was 120 and 60 cm, respectively. Weeds were removed by a minirototiller between rows and manually within rows during plant establishment. Established sugarcane plots were monitored for disease symptoms and insect damage (Table S2). Symptoms of sugarcane orange rust and elongated red lesion on midrib (red rot) were visually inspected and severity of symptoms scored using a rating scale of 0–4 as described in Glynn *et al*. (2013). 0 represents no disease; and 4 represents completely diseased plants. Sugarcane yellow leaf virus infection was also determined by the quantitative real‐time PCR analysis (Table S2) as described below using YLV‐F and YLV‐R primers (Table S3) and normalized against glyceraldehyde 3‐phosphate dehydrogenase (*GAPDH*) as reference gene. For the control of insects such as mealybugs, scales, and aphids, Bifenthrin (Brigade^®^ 2EC), Imidacloprid (Admire^®^ Pro) or Sulfoxaflor (Transform™) were applied at the labeled rate. For the control of orange rust, Pyraclostrobin (Headline^®^) was applied at the labeled rate. Plots were fertilized with 34 kg/ha N, 11 kg/ha P, and 34 kg/ha K at planting and 1 month after planting and irrigated daily with a rate of 10 mm for 15 days following transplanting. During the grand growth period, plots were fertilized twice in intervals of 6 weeks with 85 kg/ha N, 20 kg/ha P, and 85 kg/ha K. The established plots were irrigated up to three times a week depending on rainfall to provide at least 30 mm of irrigation per week.

One month before harvest *COMT* mutant lines and control plants were evaluated for plant height (length from base of the plant to shoot apical meristem), number of tillers per plant, and stalk diameter (middle of the stalk). Three plants per row were measured for each agronomic trait and means derived for statistical analysis. Plots were harvested on October 26, 2015 for determination of biomass, juice volume, and Brix (soluble solids). For biomass weight determination, all plants per row were harvested and the aboveground fresh biomass weight measured. To determine dry weight of the biomass, a subsample was taken from each row consisting of two mature stalks. These were chopped into small pieces, the fresh weight was determined and the biomass was dried at 60 °C for 4 weeks until constant weight was reached and the dry weight was determined. Biomass dry weight of mutant lines and controls was calculated by multiplication of the fresh biomass weight with the ratio of each subsample dry weight/fresh weight.

### Quantitative real‐time RT‐PCR analysis

Top visible dewlap leaf was collected for analysis of the eight *COMT* mutant lines, one RNAi control and WT from each of the three replicated plots. Total RNA was isolated using TRIzol reagent (Invitrogen, Grand Island, NY) and treated with RNase‐Free RQ1 DNase (Promega, San Luis Obispo, CA) according to the manufacturer's instructions. cDNA was synthesized from 1 μg of RNA using iScript cDNA synthesis kit (Bio‐Rad, Hercules, CA). The sugarcane glyceraldehyde 3‐phosphate dehydrogenase (*GAPDH*) primers were used to amplify a *GAPDH* gene fragment as a reference for normalization of transcripts as described by Iskandar *et al*. (2004). *COMT* target site was amplified using COMT_EF1 and COMT_ER1 primers (Table S3). Quantitative real‐time PCR of the transcripts was performed in the CFX Connect Real‐Time PCR (Bio‐Rad, Hercules, CA) with SsoAdvanced SYBR Green Supermix (Bio‐Rad) under the following conditions: 95 °C for 3 min denaturation, 40 cycles at 95 °C for 10 s, and 58 °C for 45 s. Amplification specificity was verified by melt curve analysis from 55 to 95 °C. COMT expression levels in mutant and RNAi plants relative to WT were calculated using the $2^{-\Delta \Delta C_{t}}$ method (Livak and Schmittgen, 2001).

### Capillary electrophoresis

Genomic DNA was extracted from 150 mg leaf tissues of field‐grown *COMT* mutant lines and WT using CTAB method (Murray and Thompson, 1980). After DNA quantification, 100 ng of DNA was used as a template for PCR. PCR fragment encompassing the TALEN target site was amplified using 4F and 6‐FAM dye‐labeled 128R primers (Table S3). PCR was performed with Q5 High‐Fidelity DNA Polymerase (NEB, Ipswich, MA) under the following conditions: 98 °C for 30 s, 34 cycles of amplification at 98 °C for 10 s, 72 °C for 10 s, and 72 °C for 10 s, and final extension at 72 °C for 2 min. Capillary electrophoresis of the PCR amplicon was performed by GENEWIZ (South Plainfield, NJ) using Applied Biosystems 3730xl Genetic Analyzer (Life Technologies, Grand Island, NY). Electropherogram was analyzed using the Peak Scanner Software v2.0 (Life Technologies). A peak at 125 bp was considered as WT *COMT*, and peaks other than 125 bp were analyzed as mutant *COMT*s. Mutation frequency in the *COMT* amplicon population was estimated by quantifying relative fluorescence of the electropherogram peaks. Mutation frequency is the ratio of sum of peak height of all mutant peaks to sum of peak height of all mutant peaks plus WT peak and expressed as percentage.

### Lignin and cell wall carbohydrates

Field‐grown matured sugarcane stalks were harvested from each plot and crushed with a roller mill to extract juice. The bagasse of each accession was dried at 45 °C until constant weight and ground using a Wiley mill (Thomas Scientific) with 2.0‐mm sieve. Two grams of ground sample was washed three times with 50% ethanol and one time with distilled water while incubated at 45 °C for 1 h during each washing step. The washed samples were dried at 45 °C for 1 day and passed through a 0.42‐mm sieve. The sample was further dried at 45 °C until constant weight. Total lignin contents were determined using the modified acetyl bromide method as described by Jung *et al*. (2012). The lignin content was calculated using sugarcane vascular bundle lignin molar extinction coefficient of 21.5 L/g/cm as reported in He and Terashima (1990).

Lignin compositions were determined using a pyrolysis molecular beam mass spectrometer. Each sample was prepared in triplicate by weighing about 1.0–2.5 mg into a stainless metal cup and pyrolyzed at 500 °C to produce volatile compounds. The volatile compounds were analyzed using a molecular beam mass spectrometer (Extrel Pittsburgh, PA). NIST 8492 (National Institute of Standards and Technology) lignin content 26.2% was used as a lignin standard (Jung *et al*., 2016).

Cell wall carbohydrates were analyzed using the National Renewable Energy Laboratory (NREL) protocol by Sluiter *et al*. (2008). Liberated monomeric sugars were identified and quantified with an Agilent/HP 1200 HPLC equipped with an RI detector (Agilent Technologies, Santa Clara, CA). The HPLC analysis was carried out using a Hi‐Plex H column (Agilent Technologies), operating at a flow rate of 0.6 mL/min using 5 mm H_2_SO_4_ as a mobile phase.

### Enzymatic hydrolysis

Dilute acid pretreatment and enzymatic hydrolysis were performed as previously described (Jung *et al*., 2013). The enzymatic hydrolysis was performed for 72 h. Glucose from enzymatic hydrolysis was measured by an Agilent 1200 series modular HPLC with RI detector (Agilent Technologies). The HPLC analysis was carried out using a HPX‐87H column (Bio‐Rad), operating at a flow rate of 0.6 mL/min using 5 mm H_2_SO_4_ as a mobile phase. Saccharification efficiency was calculated as the ratio of glucose released following the enzymatic hydrolysis to the amount of glucose present in the cell wall before the hydrolysis.

### Statistical analysis

Gene expression, total lignin content, cell wall carbohydrate content, saccharification efficiency, agronomic trait components and biomass yield data were statistically analyzed. *T*‐tests were performed using SAS™ version 9.3 (SAS Institute Inc., Cary, CA) to determine whether the means were significantly different between mutant lines and control plants (*n *=* *3, *P *<* *0.05).

## Supporting information


**Figure S1** Quantitative RT‐PCR analysis of *COMT* expression in field grown, TALEN mediated *COMT* mutants and control plants
**Figure S2** (a) DNA sequences of TALEN binding and target sites in the first exon of the sugarcane *COMT*. P^1^‐P^2^ and P^1^‐P^3^ are primer binding sites for amplification of TALEN target site or long PCR amplicons for *COMT* copy/allele identification, respectively. (b) Sequence confirmation of TALEN mediated *COMT* mutation in PCR amplicons. Deletions ranging from 2 to 48 bp in one of the mutants (CB6) considered as different mutation types.
**Figure S3** Schematic representation of the region of *COMT* that was PCR amplified, cloned and sequenced for identification of mutations in different *COMT* copies/alleles by the Sanger method.

**Table S1** Number of copies/alleles of *COMT* and types of TALEN mediated target mutation in sugarcane.
**Table S2** Disease symptoms and insect damage on COMT mutant, RNAi/suppressed sugarcane, WT and transgenic control plants.
**Table S3** A list of primer pairs used in this study.

**Data S1** Supplementary Experimental Procedures and Results.
