# Supplementary material for: TALEN‐mediated targeted mutagenesis of more than 100 COMT copies/alleles in highly polyploid sugarcane improves saccharification efficiency without compromising biomass yield
Source: Plant Biotechnol J. 2017 Nov 18;16(4):856–66. doi: 10.1111/pbi.12833 (PMC5866949; doi:10.1111/pbi.12833)
Supplement: Supplementary file 1 — Figure S1 Quantitative RT‐PCR analysis of COMT expression in field grown, TALEN mediated COMT mutants and control plants Figure S2 (a) DNA sequences of TALEN binding and target sites in the first exon of the sugarcane COMT. P1‐P2 and P1‐P3 are primer binding sites for amplification of TALEN target site or long PCR amplicons for COMT copy/allele identification, respectively. (b) Sequence confirmation of TALEN mediated COMT mutation in PCR amplicons. Deletions ranging from 2 to 48 bp in one of the mutants (CB6) considered as different mutation types. Figure S3 Schematic representation of the region of COMT that was PCR amplified, cloned and sequenced for identification of mutations in different COMT copies/alleles by the Sanger method. [file PBI-16-856-s003.pptx]

## Slide 1
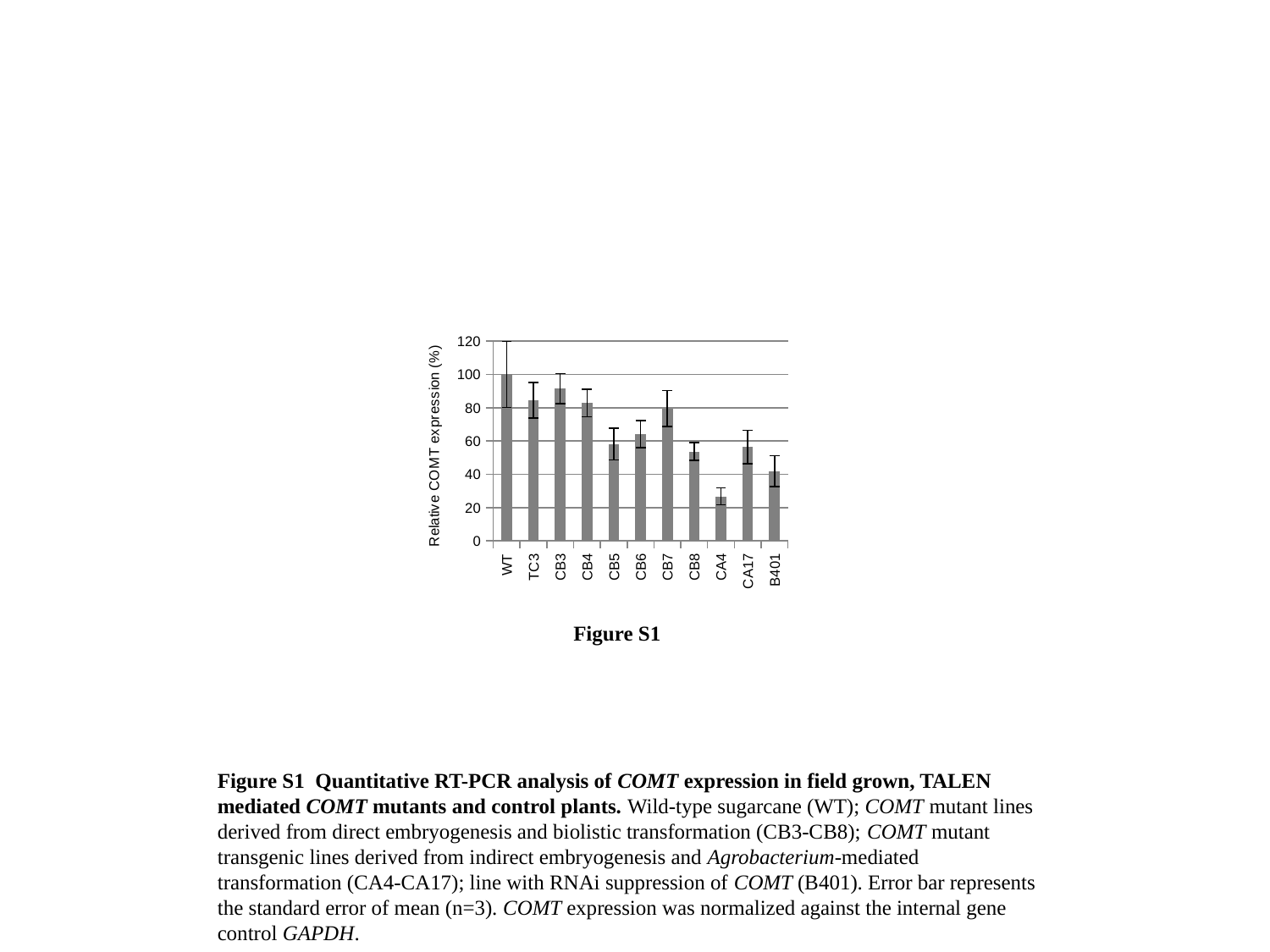

### Chart
| Category | |
|---|---|
| WT | 100.00000000000001 |
| TC3 | 84.40571001110045 |
| CB3 | 91.39165474291335 |
| CB4 | 82.84548117426212 |
| CB5 | 58.083481728934395 |
| CB6 | 64.25186275551825 |
| CB7 | 79.4671291518523 |
| CB8 | 53.71384954415133 |
| CA4 | 26.760916766911098 |
| CA17 | 56.41572268695101 |
| B401 | 41.860474260819366 |Figure S1
Figure S1 Quantitative RT-PCR analysis of COMT expression in field grown, TALEN mediated COMT mutants and control plants. Wild-type sugarcane (WT); COMT mutant lines derived from direct embryogenesis and biolistic transformation (CB3-CB8); COMT mutant transgenic lines derived from indirect embryogenesis and Agrobacterium-mediated transformation (CA4-CA17); line with RNAi suppression of COMT (B401). Error bar represents the standard error of mean (n=3). COMT expression was normalized against the internal gene control GAPDH.

## Slide 2
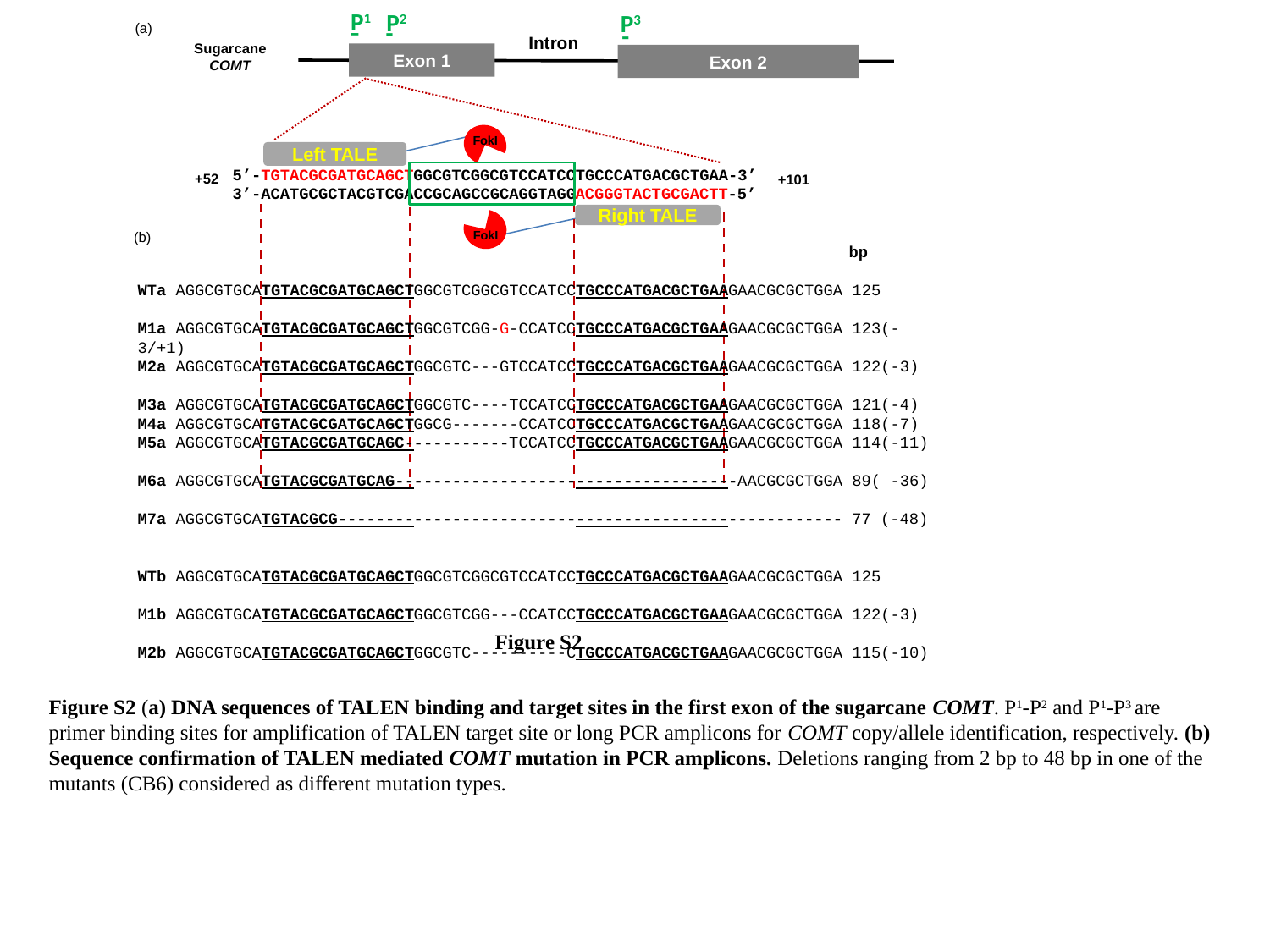

P1
P2
-
P3
-
-
Sugarcane
COMT
Intron
Exon 1
Exon 2
FokI
Left TALE
5’-TGTACGCGATGCAGCTGGCGTCGGCGTCCATCCTGCCCATGACGCTGAA-3’
3’-ACATGCGCTACGTCGACCGCAGCCGCAGGTAGGACGGGTACTGCGACTT-5’
+52
 +101
Right TALE
FokI
					 bp
WTa AGGCGTGCATGTACGCGATGCAGCTGGCGTCGGCGTCCATCCTGCCCATGACGCTGAAGAACGCGCTGGA 125
M1a AGGCGTGCATGTACGCGATGCAGCTGGCGTCGG-G-CCATCCTGCCCATGACGCTGAAGAACGCGCTGGA 123(-3/+1)
M2a AGGCGTGCATGTACGCGATGCAGCTGGCGTC---GTCCATCCTGCCCATGACGCTGAAGAACGCGCTGGA 122(-3)
M3a AGGCGTGCATGTACGCGATGCAGCTGGCGTC----TCCATCCTGCCCATGACGCTGAAGAACGCGCTGGA 121(-4)
M4a AGGCGTGCATGTACGCGATGCAGCTGGCG-------CCATCCTGCCCATGACGCTGAAGAACGCGCTGGA 118(-7)
M5a AGGCGTGCATGTACGCGATGCAGC-----------TCCATCCTGCCCATGACGCTGAAGAACGCGCTGGA 114(-11)
M6a AGGCGTGCATGTACGCGATGCAG------------------------------------AACGCGCTGGA 89( -36)
M7a AGGCGTGCATGTACGCG----------------------------------------------------- 77 (-48)
WTb AGGCGTGCATGTACGCGATGCAGCTGGCGTCGGCGTCCATCCTGCCCATGACGCTGAAGAACGCGCTGGA 125
M1b AGGCGTGCATGTACGCGATGCAGCTGGCGTCGG---CCATCCTGCCCATGACGCTGAAGAACGCGCTGGA 122(-3)
M2b AGGCGTGCATGTACGCGATGCAGCTGGCGTC----------CTGCCCATGACGCTGAAGAACGCGCTGGA 115(-10)
(a)
(b)
Figure S2
Figure S2 (a) DNA sequences of TALEN binding and target sites in the first exon of the sugarcane COMT. P1-P2 and P1-P3 are primer binding sites for amplification of TALEN target site or long PCR amplicons for COMT copy/allele identification, respectively. (b) Sequence confirmation of TALEN mediated COMT mutation in PCR amplicons. Deletions ranging from 2 bp to 48 bp in one of the mutants (CB6) considered as different mutation types.

## Slide 3
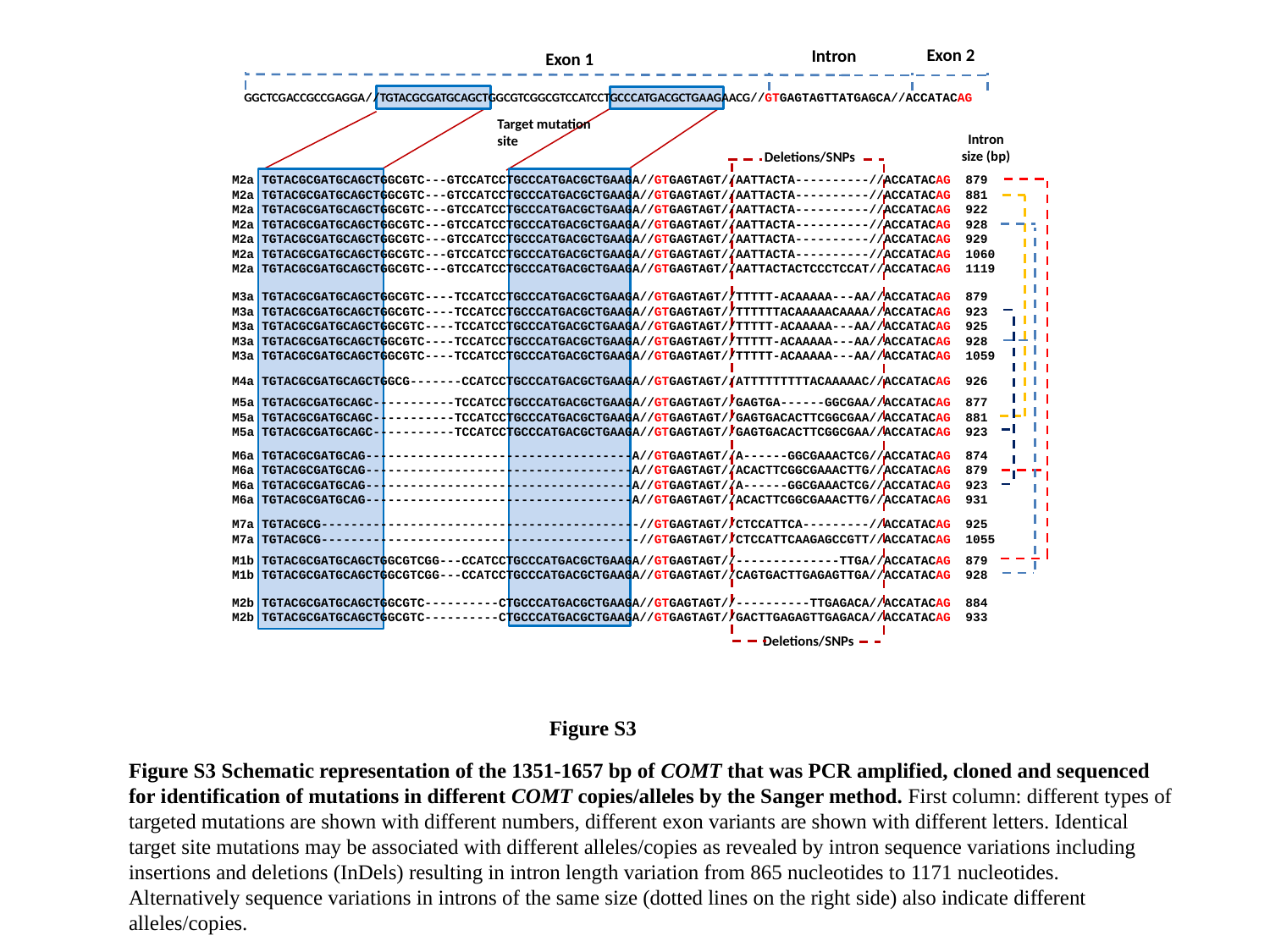

Exon 2
Intron
Exon 1
GGCTCGACCGCCGAGGA//TGTACGCGATGCAGCTGGCGTCGGCGTCCATCCTGCCCATGACGCTGAAGAACG//GTGAGTAGTTATGAGCA//ACCATACAG
Target mutation site
Intron size (bp)
Deletions/SNPs
M2a TGTACGCGATGCAGCTGGCGTC---GTCCATCCTGCCCATGACGCTGAAGA//GTGAGTAGT//AATTACTA----------//ACCATACAG 879
M2a TGTACGCGATGCAGCTGGCGTC---GTCCATCCTGCCCATGACGCTGAAGA//GTGAGTAGT//AATTACTA----------//ACCATACAG 881
M2a TGTACGCGATGCAGCTGGCGTC---GTCCATCCTGCCCATGACGCTGAAGA//GTGAGTAGT//AATTACTA----------//ACCATACAG 922
M2a TGTACGCGATGCAGCTGGCGTC---GTCCATCCTGCCCATGACGCTGAAGA//GTGAGTAGT//AATTACTA----------//ACCATACAG 928
M2a TGTACGCGATGCAGCTGGCGTC---GTCCATCCTGCCCATGACGCTGAAGA//GTGAGTAGT//AATTACTA----------//ACCATACAG 929
M2a TGTACGCGATGCAGCTGGCGTC---GTCCATCCTGCCCATGACGCTGAAGA//GTGAGTAGT//AATTACTA----------//ACCATACAG 1060
M2a TGTACGCGATGCAGCTGGCGTC---GTCCATCCTGCCCATGACGCTGAAGA//GTGAGTAGT//AATTACTACTCCCTCCAT//ACCATACAG 1119
M3a TGTACGCGATGCAGCTGGCGTC----TCCATCCTGCCCATGACGCTGAAGA//GTGAGTAGT//TTTTT-ACAAAAA---AA//ACCATACAG 879
M3a TGTACGCGATGCAGCTGGCGTC----TCCATCCTGCCCATGACGCTGAAGA//GTGAGTAGT//TTTTTTACAAAAACAAAA//ACCATACAG 923
M3a TGTACGCGATGCAGCTGGCGTC----TCCATCCTGCCCATGACGCTGAAGA//GTGAGTAGT//TTTTT-ACAAAAA---AA//ACCATACAG 925
M3a TGTACGCGATGCAGCTGGCGTC----TCCATCCTGCCCATGACGCTGAAGA//GTGAGTAGT//TTTTT-ACAAAAA---AA//ACCATACAG 928
M3a TGTACGCGATGCAGCTGGCGTC----TCCATCCTGCCCATGACGCTGAAGA//GTGAGTAGT//TTTTT-ACAAAAA---AA//ACCATACAG 1059
M4a TGTACGCGATGCAGCTGGCG-------CCATCCTGCCCATGACGCTGAAGA//GTGAGTAGT//ATTTTTTTTTACAAAAAC//ACCATACAG 926
M5a TGTACGCGATGCAGC-----------TCCATCCTGCCCATGACGCTGAAGA//GTGAGTAGT//GAGTGA------GGCGAA//ACCATACAG 877
M5a TGTACGCGATGCAGC-----------TCCATCCTGCCCATGACGCTGAAGA//GTGAGTAGT//GAGTGACACTTCGGCGAA//ACCATACAG 881
M5a TGTACGCGATGCAGC-----------TCCATCCTGCCCATGACGCTGAAGA//GTGAGTAGT//GAGTGACACTTCGGCGAA//ACCATACAG 923
M6a TGTACGCGATGCAG------------------------------------A//GTGAGTAGT//A------GGCGAAACTCG//ACCATACAG 874
M6a TGTACGCGATGCAG------------------------------------A//GTGAGTAGT//ACACTTCGGCGAAACTTG//ACCATACAG 879
M6a TGTACGCGATGCAG------------------------------------A//GTGAGTAGT//A------GGCGAAACTCG//ACCATACAG 923
M6a TGTACGCGATGCAG------------------------------------A//GTGAGTAGT//ACACTTCGGCGAAACTTG//ACCATACAG 931
M7a TGTACGCG-------------------------------------------//GTGAGTAGT//CTCCATTCA---------//ACCATACAG 925
M7a TGTACGCG-------------------------------------------//GTGAGTAGT//CTCCATTCAAGAGCCGTT//ACCATACAG 1055
M1b TGTACGCGATGCAGCTGGCGTCGG---CCATCCTGCCCATGACGCTGAAGA//GTGAGTAGT//--------------TTGA//ACCATACAG 879
M1b TGTACGCGATGCAGCTGGCGTCGG---CCATCCTGCCCATGACGCTGAAGA//GTGAGTAGT//CAGTGACTTGAGAGTTGA//ACCATACAG 928
M2b TGTACGCGATGCAGCTGGCGTC----------CTGCCCATGACGCTGAAGA//GTGAGTAGT//----------TTGAGACA//ACCATACAG 884
M2b TGTACGCGATGCAGCTGGCGTC----------CTGCCCATGACGCTGAAGA//GTGAGTAGT//GACTTGAGAGTTGAGACA//ACCATACAG 933
Deletions/SNPs
Figure S3
Figure S3 Schematic representation of the 1351-1657 bp of COMT that was PCR amplified, cloned and sequenced for identification of mutations in different COMT copies/alleles by the Sanger method. First column: different types of targeted mutations are shown with different numbers, different exon variants are shown with different letters. Identical target site mutations may be associated with different alleles/copies as revealed by intron sequence variations including insertions and deletions (InDels) resulting in intron length variation from 865 nucleotides to 1171 nucleotides. Alternatively sequence variations in introns of the same size (dotted lines on the right side) also indicate different alleles/copies.
