# Supplementary material for: TALEN‐mediated targeted mutagenesis of more than 100 COMT copies/alleles in highly polyploid sugarcane improves saccharification efficiency without compromising biomass yield
Source: Plant Biotechnol J. 2017 Nov 18;16(4):856–66. doi: 10.1111/pbi.12833 (PMC5866949; doi:10.1111/pbi.12833)
Supplement: Supplementary file 2 — Table S1 Number of copies/alleles of COMT and types of TALEN mediated target mutation in sugarcane. Table S2 Disease symptoms and insect damage on COMT mutant, RNAi/suppressed sugarcane, WT and transgenic control plants. Table S3 A list of primer pairs used in this study. [file PBI-16-856-s002.docx]

**Table S1.** Number of copies/alleles of *COMT* and types of TALEN mediated target mutation in sugarcane.

| COMT copy/  allele | Plasmid name (s) | Target mutation type† | Plasmid name | Target mutation type | Size of sequenced insert | Intron size | Total number of long reads |
| --- | --- | --- | --- | --- | --- | --- | --- |
| 1 | 6-10 | 4a, NS | 126 | 7a, SI | 1351 | 865 | 2 |
| 2 | 23, 69, 74, 114, 225 | 4a, NS | 110 | 7a, SI | 1351 | 865 | 6 |
| 3 | 117 | 7a, SI |  |  | 1353 | 867 | 1 |
| 4 | 47 | 3a, NS |  |  | 1354 | 868 | 1 |
| 5 | 360 | 3a, NS |  |  | 1354 | 868 | 1 |
| 6 | 24 | 4a, NS |  |  | 1355 | 869 | 1 |
| 7 | 35 | 7a, SI |  |  | 1355 | 869 | 1 |
| 8 | 102 | 4a, NS |  |  | 1355 | 869 | 1 |
| 9 | 289 | 3a, NS |  |  | 1355 | 869 | 1 |
| 10 | 6-60 | 7a, SI |  |  | 1356 | 870 | 1 |
| 11 | 125 | 3a, NS |  |  | 1356 | 870 | 1 |
| 12 | 201, 253 | 3a, NS |  |  | 1356 | 870 | 2 |
| 13 | 97, 174 | 2b, NS |  |  | 1356 | 870 | 2 |
| 14 | 112 | 4a, NS |  |  | 1356 | 870 | 1 |
| 15 | 206 | 2b, NS |  |  | 1356 | 870 | 1 |
| 16 | 115 | 3a, NS |  |  | 1357 | 871 | 1 |
| 17 | 132, 175 | 3a, NS |  |  | 1357 | 871 | 2 |
| 18 | 156 | 2b, NS |  |  | 1357 | 871 | 1 |
| 19 | 6-86 | 7a, SI |  |  | 1359 | 873 | 1 |
| 20 | 6-90 | 2b, NS |  |  | 1359 | 873 | 1 |
| 21 | 41 | 1a, MS |  |  | 1359 | 873 | 1 |
| 22 | 45 | WT |  |  | 1359 | 873 | 1 |
| 23 | 22 | 4a, NS |  |  | 1359 | 873 | 1 |
| 24 | 370 | 7a, SI |  |  | 1359 | 873 | 1 |
| 25 | 6-2, 6-50, 6-51, 6-63, 6-76, 3, 9, 10, 20, 48, 61, 64, 100, 150, 185, 191, 223, 229, 250, 267, 297, 298, 302, 311, 315, 322, 349, 359, 368 | 1b, SI |  |  | 1361 | 875 | 29 |
| 26 | 325 | 7a, SI |  |  | 1361 | 875 | 1 |
| 27 | 358 | 7a, SI |  |  | 1361 | 875 | 1 |
| 28 | 109 | 2b, NS |  |  | 1363 | 877 | 1 |
| 29 | 63 | 1b, SI |  |  | 1365 | 879 | 1 |
| 30 | 52 | 6a, SI |  |  | 1397 | 911 | 1 |
| 31 | 68 | 2b, NS |  |  | 1397 | 911 | 1 |
| 32 | 172 | 3a, NS |  |  | 1397 | 911 | 1 |
| 33 | 178 | 3a, NS |  |  | 1397 | 911 | 1 |
| 34 | 123 | 4a, NS |  |  | 1397 | 911 | 1 |
| 35 | 2 | 7a, SI |  |  | 1399 | 913 | 1 |
| 36 | 319 | 7a, SI |  |  | 1399 | 913 | 1 |
| 37 | 6-79, 342 | 3a, NS |  |  | 1400 | 914 | 2 |

**Table S1.** Contd.,

| COMT copy/ allele | Plasmid name (s) | Target mutation type† | Plasmid name (s) | Target mutation type | Plasmid name | Target mutation type | Size of sequenced insert | Intron size | Total number of long reads |
| --- | --- | --- | --- | --- | --- | --- | --- | --- | --- |
| 38 | 6-4, 6-7, 6-8, 6-58, 6-75, 6-77, 6-84, 6-85, 6-94, 15, 16, 18, 33, 34, 37, 39, 42, 53, 59, 62, 75, 78, 95, 99, 105, 113, 118, 124, 131, 141, 144, 152, 157, 162, 168, 170, 176, 177, 179, 188, 202, 207, 221, 236, 247, 263, 265, 266, 272, 277, 279, 280, 286, 293, 307, 308, 310, 312, 335, 352, 355, 356, 365, 366, 369, 381, 383 | 4a, NS | 6-73, 84, 96 | 7a, SI | **6-67** (exon var.)‡ | 1b, SI | 1400 | 914 | 71 |
| 39 | 6-3, 6-62, 6-78, 6-83, 6-89, 11, 12, 19, 26, 36, 38, 43, 51, 54, 55, 60, 66, 67, 73, 86, 103, 119, 127, 135, 142, 143, 146, 147, 181, 189, 192, 193, 203, 213, 217, 227, 237, 258, 259, 261, 270, 288, 295, 299, 314, 316, 317, 323, 324, 326, 329, 334, 339, 345, 348, 353, 363, 367, 379, 380, [**330** (exon var.)] | 7a, SI | 40, 116, 128 | 4a, NS | **71** (exon var.) | 1a, MS | 1400 | 914 | 65 |
| 40 | 30, 166 | 3a, NS |  |  |  |  | 1400 | 914 | 2 |
| 41 | 101, 121, 159, 183, 343 | 4a, NS | **208** (exon var.) | 2b, NS |  |  | 1400 | 914 | 6 |
| 42 | 246 | 4a, NS |  |  |  |  | 1400 | 914 | 1 |
| 43 | 154 | 2b, NS |  |  |  |  | 1400 | 914 | 1 |
| 44 | 190 | 2b, NS | 249 | 1b, SI |  |  | 1400 | 914 | 2 |
| 45 | 104, 136, 173, 212, 271, 281, 290, 364 | 7a, SI |  |  |  |  | 1400 | 914 | 8 |
| 46 | 158 | 6a, SI |  |  |  |  | 1400 | 914 | 1 |
| 47 | 32, 50, 87, 98, 151, 199, 219, 282, 305, 320 | 4a, NS |  |  |  |  | 1401 | 915 | 10 |
| 48 | 56 | 4a, NS |  |  |  |  | 1401 | 915 | 1 |

**Table S1.** Contd.,

| COMT copy/ allele | Plasmid name (s) | Target mutation type† | Plasmid name (s) | Target mutation type | Plasmid name | Target mutation type | Size of sequenced insert | Intron size | Total number of long reads |
| --- | --- | --- | --- | --- | --- | --- | --- | --- | --- |
| 49 | 28, 58, 211 | 4a, NS | 27, 46, 92, 138, 148, 164, 304 | 7a, SI | 111 | 6a, SI | 1401 | 915 | 11 |
| 50 | 6-57 | 2a, SI |  |  |  |  | 1402 | 916 | 1 |
| 51 | 65 | 4a, NS |  |  |  |  | 1402 | 916 | 1 |
| 52 | 88 | 7a, SI |  |  |  |  | 1402 | 916 | 1 |
| 53 | 122 | 1b, SI |  |  |  |  | 1402 | 916 | 1 |
| 54 | 336 | 1b, SI |  |  |  |  | 1402 | 916 | 1 |
| 55 | 5 | 4a, NS |  |  |  |  | 1402 | 916 | 1 |
| 56 | 222 | 4a, NS |  |  |  |  | 1402 | 916 | 1 |
| 57 | 283 | 4a, NS |  |  |  |  | 1402 | 916 | 1 |
| 58 | 241 | 7a, SI |  |  |  |  | 1402 | 916 | 1 |
| 59 | 284 | 7a, SI |  |  |  |  | 1402 | 916 | 1 |
| 60 | 44 | 7a, SI |  |  |  |  | 1403 | 917 | 1 |
| 61 | 6-53 | 3a, NS |  |  |  |  | 1404 | 918 | 1 |
| 62 | 13 | 2b, NS |  |  |  |  | 1404 | 918 | 1 |
| 63 | 57 | 3a, NS |  |  |  |  | 1404 | 918 | 1 |
| 64 | 341 | 3a, NS |  |  |  |  | 1404 | 918 | 1 |
| 65 | 145 | 2b, NS |  |  |  |  | 1404 | 918 | 1 |
| 66 | 300 | 2b, NS |  |  |  |  | 1404 | 918 | 1 |
| 67 | 6-59, 6-64, 6-66, 6-80, 6-82, 6-87, 4, 8, 14, 49, 77, 153, 165, 169, 182, 184, 186, 195, 197, 215, 218, 228, 234, 239, 244, 245, 254, 264, 269, 287, 294, 328, 338, 346, 361, 371, 372, 373, 384 | 3a, NS |  |  |  |  | 1405 | 919 | 39 |
| 68 | 6-72, 6-74, 6-81, 6-91, 89, 90, 106, 134, 194, 205, 230, 231, 240, 243, 255, 257, 275, 303, 331, 350, 362, 374, 377, 378 | 2b, NS |  |  |  |  | 1405 | 919 | 24 |
| 69 | 6-65 | 2b, NS |  |  |  |  | 1405 | 919 | 1 |
| 70 | 6-68 | 6a, SI |  |  |  |  | 1405 | 919 | 1 |

**Table S1.** Contd.,

| COMT copy/ allele | Plasmid name (s) | Target mutation type† | Plasmid name (s) | Target mutation type | Size of sequenced insert | Intron size | Total number of long reads |
| --- | --- | --- | --- | --- | --- | --- | --- |
| 71 | 6-93, 1 | 7a, SI | **6-49**, **6-52** (exon var.) | 2b, NS | 1405 | 919 | 4 |
| 72 | 17 | 2b, NS |  |  | 1405 | 919 | 1 |
| 73 | 25 | 7a, SI |  |  | 1405 | 919 | 1 |
| 74 | 91 | 3a, NS |  |  | 1405 | 919 | 1 |
| 75 | 94 | 6a, SI |  |  | 1405 | 919 | 1 |
| 76 | 70 | WT |  |  | 1405 | 919 | 1 |
| 77 | 139 | 3a, NS |  |  | 1405 | 919 | 1 |
| 78 | 232, 256 | 3a, NS |  |  | 1405 | 919 | 2 |
| 79 | 375 | 2b, NS |  |  | 1405 | 919 | 1 |
| 80 | 376 | 2b, NS |  |  | 1405 | 919 | 1 |
| 81 | 318 | 7a, SI |  |  | 1405 | 919 | 1 |
| 82 | 351 | 4a, NS |  |  | 1405 | 919 | 1 |
| 83 | 107 | 3a, NS |  |  | 1406 | 920 | 1 |
| 84 | 21, 129, 248 | 3a, NS |  |  | 1406 | 920 | 3 |
| 85 | 233, 276 | 3a, NS |  |  | 1406 | 920 | 2 |
| 86 | 200 | 4a, NS |  |  | 1406 | 920 | 1 |
| 87 | 161, 198 | 2b, NS |  |  | 1406 | 920 | 2 |
| 88 | 285 | 2b, NS |  |  | 1406 | 920 | 1 |
| 89 | 220 | 2b, NS |  |  | 1406 | 920 | 1 |
| 90 | 340 | 4a, NS |  |  | 1408 | 922 | 1 |
| 91 | 216 | 1b, SI |  |  | 1409 | 923 | 1 |
| 92 | 6-92, 85 | 1b, SI |  |  | 1410 | 924 | 2 |
| 93 | 140 | 3b, SI |  |  | 1410 | 924 | 1 |
| 94 | 274, 278 | 1b, SI |  |  | 1410 | 924 | 2 |
| 95 | 72 | 1b, SI |  |  | 1411 | 925 | 1 |
| 96 | 327 | 1b, SI |  |  | 1411 | 925 | 1 |
| 97 | 187 | 3a, NS |  |  | 1411 | 925 | 1 |
| 98 | 120 | 1b, SI |  |  | 1550 | 1064 | 1 |
| 99 | 6-1, 6-6 | 1a, MS |  |  | 1594 | 1108 | 2 |
| 100 | 6-88, 7, 93, 137, 260 | 6a, SI |  |  | 1651 | 1165 | 5 |
| 101 | 321, 332, 344 | 6a, SI |  |  | 1651 | 1165 | 3 |
| 102 | 209 | 6a, SI |  |  | 1651 | 1165 | 1 |
| 103 | 224 | 4a, NS |  |  | 1651 | 1165 | 1 |
| 104 | 6-71 | 2b, NS |  |  | 1657 | 1171 | 1 |

^†^ Mutation type:

WT, no nucleotide deletion/addition in target site;

1a, 3 bp deletion and 1 bp addition, MS, Missense mutation (Frame shift)

2a, 3 bp deletion, SI, silent mutation (one codon deletion)

3a, 4 bp deletion, NS, nonsense mutation (Frame shift leads to early stop codon)

4a, 7 bp deletion, NS, nonsense mutation (Frame shift leads to early stop codon)

6a, 36 bp deletion, SI, silent mutation (12 codon deletion)

7a, 48 bp deletion, SI, silent mutation (16 codon deletion)

1b, 3 bp deletion, SI, silent mutation (one codon deletion)

2b, 10 bp deletion, NS, nonsense mutation (Frame shift leads to early stop codon)

3b, 7 bp deletion and 4 bp addition, SI, silent mutation

^‡^ Exon var., exon variant with at least one SNP in the first exon outside of the targeted mutation site, bold letters highlight unique reads with exon variation

**Table S2.** Disease symptoms and insect damage on *COMT* mutant, RNAi/suppressed sugarcane, WT and transgenic control plants.

| Lines | Disease symptoms ^a^ | | | | Normalized expression of SCYLV in qRT-PCR | Insect damage ^a†^ |
| --- | --- | --- | --- | --- | --- | --- |
|  | Red rot ^†^ | Orange rust ^†^ | Red rot ^‡^ | Orange rust ^‡^ |  |  |
| WT | 1.00 ± 0.07 | 0.50 ± 0.00 | 0.50 ± 0.00 | 0.50 ± 0.00 | 0.000 | 0.00 |
| TC1 | 0.05 ± 0.00 | 0.50 ± 0.00 | 0.50 ± 0.00 | 0.50 ± 0.00 | nd | 0.00 |
| TC2 | 1.00 ± 0.00 | 0.75 ± 0.20 | 0.50 ± 0.00 | 0.50 ± 0.00 | nd | 0.00 |
| TC4 | 1.00 ± 0.00 | 0.50 ± 0.00 | 0.50 ± 0.00 | 0.50 ± 0.00 | 0.000 | 0.00 |
| CB3 | 0.83 ± 0.17 | 0.50 ± 0.00 | 0.50 ± 0.00 | 0.50 ± 0.00 | 0.000 | 0.00 |
| CB4 | 0.83 ± 0.17 | 0.50 ± 0.00 | 0.50 ± 0.00 | 0.50 ± 0.00 | 0.000 | 0.00 |
| CB5 | 1.00 ± 0.00 | 0.50 ± 0.00 | 0.50 ± 0.00 | 0.50 ± 0.00 | 0.000 | 0.00 |
| CB6 | 1.00 ± 0.00 | 0.50 ± 0.00 | 0.50 ± 0.00 | 0.50 ± 0.00 | 0.000 | 0.00 |
| CB7 | 0.83 ± 0.17 | 0.50 ± 0.00 | 0.50 ± 0.00 | 0.50 ± 0.00 | 0.000 | 0.00 |
| CB8 | 0.67 ± 0.17 | 0.67 ± 0.17 | 0.50 ± 0.00 | 0.50 ± 0.00 | 0.000 | 0.00 |
| CA4 | 0.67 ± 0.17 | 0.50 ± 0.00 | 0.33 ± 0.08 | 0.50 ± 0.00 | 0.000 | 0.00 |
| CA17 | 0.67 ± 0.17 | 0.83 ± 0.17 | 0.33 ± 0.17 | 0.58 ± 0.08 | 0.000 | 0.00 |
| B401 | 1.00 ± 0.00 | 0.50 ± 0.00 | 0.42 ± 0.08 | 0.42 ± 0.08 | 0.000 | 0.00 |
| WT-I ^§^ | nd | nd | nd | nd | 1.821 | nd |

WT, wild-type sugarcane; TC1, callus derived control harboring the *npt*II gene; TC2, callus derived non-transgenic control; TC4, direct embryogenesis derived transgenic control with no mutation; CB3-CB8, transgenic lines derived from biolistic transformation, CA4-CA17, transgenic lines derived from *Agrobacterium*-mediated transformation; 2B101, B401, *COMT* RNAi lines.

Values are means ± standard error of the mean.

^a^ Scored as 0-4, 0 represents no disease or pest damage and 4 represents completely diseased or damaged. Elongated red lesion on the sugarcane leaf midrib was scored as red rot.

^†^ Scored on 31 July 2015; ^‡^ Scored on 4 Sep 2015

^§^ WT-I, sugarcane CP88-1762 infected with sugarcane yellow leaf virus (SCYLV) used as a positive control for the determination of infection in other lines and WT in qRT-PCR. SCYLV expression was normalized against the internal gene control *GAPDH*

nd - not determined

**Table S3.** A list of primer pairs used in this study

| Purpose | Primer name | Primer sequence (5′ → 3′) |
| --- | --- | --- |
| *COMT* mutant site screening | 4F (P1) | GGCTCGACCGCCGAGGAC |
|  | 128R (P2) | TCCAGCAGGCCCAGCTCCAG |
| Capillary electrophoresis  of amplicon | 4F (P1) | GGCTCGACCGCCGAGGAC |
|  | [6-FAM] 128R (P2) | [6-FAM] TCCAGCAGGCCCAGCTCCAG |
| *COMT* gene expression | COMT_EF1 | ACGCCATCCTCATGAAGTG |
|  | COMT_ER1 | CGTAGCAGTTCTTGAGCAG |
| *COMT* long amplicon for Sanger sequencing | 4F (P1) | GGCTCGACCGCCGAGGAC |
|  | BK_RO7 (P3) | CGTCATCCCGTACGCCTTGTTG |
| SCYLV infection screening | ScYLVf1 | GACAGACTCGGCCAGTGGTCGTG |
|  | ScYLVr1 | GTAAGCCATTGTTGAACGCTGCG |
