# Supplementary material for: TALEN‐mediated targeted mutagenesis of more than 100 COMT copies/alleles in highly polyploid sugarcane improves saccharification efficiency without compromising biomass yield
Source: Plant Biotechnol J. 2017 Nov 18;16(4):856–66. doi: 10.1111/pbi.12833 (PMC5866949; doi:10.1111/pbi.12833)
Supplement: Supplementary file 3 — Data S1 Supplementary Experimental Procedures and Results. [file PBI-16-856-s001.docx]

**Supplementary Experimental Procedures**

**Generation of TALEN mediated *COMT* mutant lines from sugarcane** was reported by Jung and Altpeter (2016) and is briefly summarized here.

Sugarcane *COMT* gene sequence (GenBank accession No. AJ231133) and ten tentative consensus (TC) *COMT*s were retrieved from NCBI and sugarcane EST database (DFCI *S.ofﬁcinarum* Gene Index; http://compbio.dfci. harvard.edu/cgi bin/tgi/ geneprod_search.pl,), respectively. TALEN^TM^ Hit software (<http://talen-hit.cellectis-bioresearch.com/>) was used to select TALEN binding and target sites in the first exon of the sugarcane *COMT* gene, which is a highly conserved sequence among *COMT* alleles and its putative homo(eo)logs, between 52 bp and 101 bp downstream of the translation start site (Figure S2). TALEN cassette containing CmYLCV promoter and NtHSP 3′ UTR in the left arm, CmYLCV promoter and AtHSP 3′ UTR in the right arm, selectable marker gene *npt*II containing CaMV 35s promoter and ZmHSP70 intron and CaMV 35s poly-A were introduced into the sugarcane genome using *Agrobacterium* or biolistic transformation.

Immature leaf whorl explants of sugarcane cultivar CP88-1762 grown at the Everglades Research and Education Center, University of Florida, Belle Glade, FL, were used to generate somatic embryos. Callus was used for *Agrobacterium* mediated transformation via indirect somatic embryogenesis to generate the lines designated as CA series or biolistic transformation via direct somatic embryogenesis to generate the lines designated as CB series. Following biolistic gene transfer into leaf whorl cross-sections or callus, cultures were selected on geneticin containing culture medium for seven to eight subculture cycles of 10 days or seven to 10 biweekly cycles, respectively. Regenerated plants were acclimatized in growth chamber with 80% relative humidity and 16 h photoperiod. After acclimatization, plants were transferred to greenhouse (28°C/22°C (day/night) under natural photoperiod and grown to maturity. TALEN cassette integration was confirmed by PCR screening of greenhouse grown plants. Targeted mutagenesis was confirmed by capillary electrophoresis and 454 sequencing of the 89-148 nt PCR amplicon of the highly conserved target site in the first *COMT* exon from greenhouse grown plants.

**Supplementary Results**

**Generation of TALEN mediated *COMT* mutant lines from sugarcane** was reported by Jung and Altpeter (2016) and is briefly summarized here. TALEN cassette integration into sugarcane was confirmed by PCR amplification in a total of 27 independent lines following biolistic transformation and 39 independent lines following Agrobacterium mediated transformation. Mutations of the target site were identified by capillary electrophoresis and confirmed by 454 sequencing of the 89-148 nt PCR amplicon of the highly conserved target site in the first *COMT* exon in 8 lines following biolistic transformation and 29 lines following Agrobacterium mediated transformation. TALEN mediated mutant plants were grown to maturity under greenhouse condition and altered cell wall characteristics were confirmed in greenhouse grown plants.
